# Combination of the immunization with the sequence close to the consensus sequence and two DNA prime plus one VLP boost generate H5 hemagglutinin specific broad neutralizing antibodies

**DOI:** 10.1371/journal.pone.0176854

**Published:** 2017-05-24

**Authors:** Guiqin Wang, Renfu Yin, Paul Zhou, Zhuang Ding

**Affiliations:** 1 Laboratory of Infectious Diseases, College of Veterinary Medicine, Key Laboratory of Zoonosis Research, Chinese Ministry of Education, Jilin University, Changchun, China; 2 The Unit of Anti-viral Immunity and Genetic Therapy, Key Laboratory of Molecular Virology & Immunology, Institut Pasteur of Shanghai, Chinese Academy of Sciences, Shanghai, China; Icahn School of Medicine at Mount Sinai, UNITED STATES

## Abstract

Hemagglutinin (HA) head has long been considered to be able to elicit only a narrow, strain-specific antibody response as it undergoes rapid antigenic drift. However, we previously showed that a heterologous prime-boost strategy, in which mice were primed twice with DNA encoding HA and boosted once with virus-like particles (VLP) from an H5N1 strain A/Thailand/1(KAN)-1/2004 (noted as TH DDV), induced anti-head broad cross-H5 neutralizing antibody response. To explain why TH DDV immunization could generate such breadth, we systemically compared the neutralization breadth and potency between TH DDV sera and immune sera elicited by TH DDD (three times of DNA immunizations), TH VVV (three times of VLP immunizations), TH DV (one DNA prime plus one VLP boost) and TK DDV (plasmid DNA and VLP derived from another H5N1 strain, A/Turkey/65596/2006). Then we determined the antigenic sites (AS) on TH HA head and the key residues of the main antigenic site. Through the comparison of different regiments, we found that the combination of the immunization with the sequence close to the consensus sequence and two DNA prime plus one VLP boost caused that TH DDV immunization generate broad neutralizing antibodies. Antigenic analysis showed that TH DDV, TH DV, TH DDD and TH VVV sera recognize the common antigenic site AS1. Antibodies directed to AS1 contribute to the largest proportion of the neutralizing activity of these immune sera. Residues 188 and 193 in AS1 are the key residues which are responsible for neutralization breadth of the immune sera. Interestingly, residues 188 and 193 locate in classical antigen sites but are relatively conserved among the 16 tested strains and 1,663 HA sequences from NCBI database. Thus, our results strongly indicate that it is feasible to develop broad cross-H5 influenza vaccines against HA head.

## Introduction

Highly pathogenic avian influenza (HPAI) H5 viruses of the A/goose/Guangdong/1/1996 lineage were first identified in 1996[1]. Since re-emergence in 2003, thousands of the outbreaks have occurred in poultry and wild birds in many countries. As of October 2016, 856 human infections have been confirmed, resulting in 452 deaths[2].

Vaccination is the most effective approach to control and prevent H5 influenza virus infection. However, unlike seasonal influenza which has dominant circulating strains in a given flu season, HPAI H5 viruses are co-circulating of several genetically and antigentically diverse strains from different clades and subclades. Phylogenetically, H5 HA has evolved into 10 clades from 0 to 9 and second-, third-, and fourth-order subclades[3]. The current circulating HPAI H5 viruses belong to clade 2.2, 2.3.2, 2.3.4 and 7.2[4]. To deal with such genetic and antigentic diversity of H5 viruses, it is very necessary to develop a broad influenza vaccine.

Hemagglutinin (HA) protein is the major envelope glycoprotein of influenza A virus and an attractive target for a broad influenza vaccine. HA can be divided into a head domain, composed mainly of HA1, and a stalk domain, composed of a portion of HA1 and all of HA2[5]. HA sequence analysis reveals that the stalk domain is more conserved than the head domain. Various strategies have been tested to develop a broad influenza vaccine toward the HA stalk region [6–11]. However, potential repertoire of the anti-stalk antibodies is limited. The neutralization potency is poor because dense packing of HA spikes on the virion surface impedes access to the stem [12–14]. They primarily impart protection by limiting viral spread through a cell-mediated mechanism such as ADCC. These properties suggest that it may not be wise to design broad influenza vaccines solely on stem antibodies [13]. Another approach attempts to immunize with the entire HA molecular but uses a “centralized”, consensus sequence, or a native HA which is close to the consensus sequence[5,15–17]. This vaccine strategy mitigates some of the sequence diversity between strains, particularly in the globular head region, and can be employed for protection against influenza intrasubtype viruses. This approach induces the broad neutralizing antibodies mainly against HA head, but it is not clear which domain or residues on HA head are responsible for the broad neutralizing responses.

Previously, we developed a heterologous prime-boost strategy, in which mice were primed twice with DNA plasmid encoding HA and boosted once with virus-like particles (VLP) including HA and NA from an A/Thailand/1(KAN)-1/2004 H5N1 strain, whose HA sequence is the closest to the consensus sequence among diverse WHO recommended H5 vaccine strains, noted as TH DDV [18]. TH DDV induced broad antibody responses that cross-neutralize all reported clades and subclades of HPAI H5N1 viruses and protected mice from lethal challenges by both homologous and heterologous H5N1 strains. And we determined such broadly neutralizing antibodies are mainly directed to HA head.

In the current study, we systemically compared the neutralization breadth and potency against a panel of 16 H5N1 pseudotypes covering all reported clades and subclades of HPAI H5N1 viruses between TH DDV sera and immune sera elicited by TH DDD (three times of DNA immunizations), TH VVV (three times of VLP immunizations), TH DV (one DNA prime plus one VLP boost) and TK DDV (DNA plasmid and VLP derived from another HPAI H5N1 strain A/Turkey/65596/2006). In addition, we determined the antigenic sites (AS) on TH HA recognized by TH DDV, TH DDD, TH VVV and TH DV sera, and analyzed the key residues of the main antigenic site. Through the comparison of different regiments, we found that the combination of the immunization with the sequence close to the consensus sequence and two DNA prime plus one VLP boost caused that TH DDV immunization generate broad neutralizing antibodies. We also found that the various regimens based on TH strain can induce broad neutralizing responses, TH HA specific antibodies directed to AS1 contribute to the largest proportion of the neutralization activity and residues 188 and 193 in AS1, which are relatively conserved among the 16 tested strains and 1,663 HA sequences from NCBI database, are the two key residues responsible for cross-H5 neutralization.

## Materials and methods

### Ethical statement

All animal experimental protocols were approved by the Institutional Animal Care and Use Committee at Institut Pasteur of Shanghai (Approval # A2014014) in accordance with EC directive 86/609/CEE.

### Animals

All the female BALB/c mice in the experiments were housed in micro-isolator cages ventilated under negative pressure with HEPA-filter air and a 12/12-hour light/dark cycle. Before each inoculation or euthanasia procedure, the mice were anesthetized by intraperitoneal injection of pentobarbital sodium (65 mg per kg) (Sigma) to minimize suffering. The method of the mouse euthanasia is by intraperitoneal injection of sodium pentobarbital.

### Cells

The packaging cell line 293FT was purchased from Invitrogen (Cat#R700-07, Waltham, MA USA) and maintained in complete Dulbecco modified Eagle medium [high-glucose DMEM supplemented with 10% fetal bovine serum (FBS), 2 mM L-glutamine, 1 mM sodium pyruvate, penicillin(100 U/ml), and streptomycin(100 μg/ml); Invitrogen Life Technologies]. The Madin-Darby canine kidney (MDCK) cell line (Cat# CRL-2936) and mouse fibroblast cell line L929 (Cat# CCL-1) were purchased from American Tissue Culture Company (ATCC, Manassas, VA) and maintained in complete DMEM. All these cell lines have been tested for mycoplasma contamination using MycoAlert Detection kit (LONZA, Cat# LT07-118) and were mycoplasma negative.

### Generation of H5N1 pseudotype panels

A panel of 16 H5 HA and 1 N1 NA from an A/Thailand/1(KAN)-1/2004(TH) H5N1 strain were constructed and used in this study. The H5 HA panel covers all 10 clades, 5 subclades of clade 2 and 2 subclades of clade 7 of HPAI H5N1 viruses.

Two reciprocal H5 HA head/stem chimera were prepared. It has been shown that TH DDV sera have minimum neutralization activity against an H5N1 strain A/common magpie/HongKong/5052/2007(HK5052). The chimera were prepared with overlapping PCR using codon-optimized HA encoding sequences from HK5052 and TH strains and inserted into mammalian expression vector pCMV/R. In these HA chimera the head region is composed of HA1 segment, residues 42 to 274 (according to H5 numbering), while the stem region is composed of HA2 and two segments of HA1, residues 1 to 41 and 275 to 329[19].

Five mutants based on HK5052 strain were prepared. Our previous study showed that there are four antigenic sites (AS1 to AS4) in H5 HA head region [20]. There are a total of 19 amino acid residues that differ between TH and HK5052 HA heads. 14 of 19 residues are on the HA surface and 13 of the 14 residues are scattered in AS1 to AS4 and residue 94 is outside the antigenic sites. Using HK5052 HA as a backbone, 5 HA chimera containing single AS1 to AS4 substitutions or a single mutation at position 94 from TH HA were constructed and H5N1 pseudotypes expressing these HA chimera were generated as described before[18].

Using THAS1HK5052 HA as a backbone, five additional HA chimera were constructed. Of the five mutant viruses, three have a single substitution at position 159 from S to N, 188 from A to E or 193 from K to R, one has double substitutions at positions 188 and 193 from AK to ER, and one has triple substitutions at positions 158, 159 and 160 from NST to GNA.

The methods used to generate HA and NA pseudotypes and control pseudotype expressing vesicular stomatitis virus (VSV) G protein were as described before[18]. Briefly, 4.5 x 10^6^ 293FT cells were co-transfected with 14 μg of pHR’CMV-luc, 14 μg of pCMVΔ8.2, 2 μg of pCMV/R-HA, and 0.5 μg of pCMV/R-NA using calcium phosphate precipitation. After overnight incubation, the cells were washed once with PBS and cultured in 10 ml of complete DMEM. The HA and NA pseudotype-containing supernatants were harvested after 48 hrs and stored in a -80℃ freezer in aliquots until use. The relative luciferase activity (RLA) of HA and NA pseudotype stocks were determined in MDCK cells as described before.

The amount of HA on the surface of the pseudotypes was determined by hemagglutination (HA) assay. The amount of HIV-1 gag p24 was measured by an ELISA p24 kit (ZeptoMetrix Corporation, Cat# 0801200).

### Production and quantification of H5N1 VLP

To generate TH VLP and TK VLP, 293FT packaging cells were co-transfected with packaging vector pCMV/RΔ8.2 and pCMV/R vector encoding H5 HA (TH or TK) and N1 NA using the calcium phosphate precipitation method. To generate control VLP, 293FT packaging cells were transfected only with packaging vector pCMV/RΔ8.2. The VLP-containing supernatants were harvested and loaded onto 20% sucrose cushion and ultra-centrifuged at 25,000 rpm for 2 hrs at 4°C in a Beckman centrifuge (Beckman Coulter, Fullerton, CA). The pellets were resuspended in phosphate buffered solution (PBS) and stored at -80°C in aliquots until use. The amount of HA on the surface of the VLPs was determined by HA assay [18]. The amount of HIV-1 gag p24 was measured by ELISA p24 kit according to the manufacturer’s instruction. The amount of HA on the surface of the VLPs was determined by HA assay. The ratios of HA units and the amount of gag p24 for each concentrated VLP were calculated. They were within a normal range of 2 fold as reported by Wei *et a l*[21].

### HA assay

The amount of HA proteins in all the H5N1 pseudotypes and H5N1 VLPs used in this study were measured using HA assay. Serially 2-fold diluted pseudotypes and VLPs were incubated with 0.5% red blood cells from specific-pathogenic-free (SPF) chicken at a final volume of 100 μl for 30 minutes at room temperature. The HA assays results were recorded.

### Generation of immune sera

All animal protocols were approved by the Institutional Animal Care and Use Committee at Institut Pasteur of Shanghai (Approval# A2014014). To produce immune sera, 30 female BALB/c mice (*Mus musculus*) at age of 8 weeks old were randomly divided into 5 groups (6 mice per group, S1 Table). Mice in group one were intramuscularly (i.m.) primed once with plasmid DNA and intraperitoneally (i.p.) boosted once with VLP from TH strain (TH DV). Mice in group two were i.m. primed twice with plasmid DNA and i.p. boosted once with VLP from TH strain (TH DDV). Mice in group three were i.m. immunized three times with plasmid DNA alone from TH strain (TH DDD). Mice in group four were i.p. immunized three times with VLP alone from TH strain (TH VVV). And mice in group five were i.m. primed twice with plasmid DNA and i.p. boosted once with VLP from TK strain (TK DDV). For each prime 100 μg of plasmid DNA were injected, and for each boost VLP equivalent to 512 hemagglutinin units (HAU) were injected. There was a 3-week interval between each immunization. Fourteen days after the last immunization, mice were bled. Serum samples from the same immunization groups were combined, heat inactivated at 56℃, and stored in aliquots at 4℃ until use.

### Pseudotype-based neutralization (PN) assay

PN assay was described before [22]. Briefly, MDCK cells (3 x 10^3^ cells per well) were seeded onto 96-well plate in complete DMEM overnight. About 200,000 relative luciferase activity (RLA) of the pseudotypes (S2 Table) in the HA and NA pseudotype-based neutralization assay was added. In truth, different H5HAs generate the pseudoparticles at the different efficiency. To produce 200,000 RLA, the amount of the added pseudotyes was from 5 ul to 110 ul. Their HA and Gag p24 amounts among all the pseudotypes were within a normal range of 2 fold [21]. So the reduction of RLA resulted from the interaction between the pseudotypes and neutralizing antibodies rather than something else, such as HA protein expression levels in the pseudotypes, the numbers of pseudotype particles and so on. Serially 2-fold diluted serum samples (starting at 1:40 dilution) were incubated with pseudotypes at a final volume of 100 μl at 37°C for 1 hr. Relative luciferase activity (RLA) was measured after 72 hrs by the BrightGlo Luciferase assay according to the manufacturer’s instruction (Promega, Cat#14550). Titration curves were generated using sigmoid dose-response of nonlinear fit from GraphPad and IC_50_ values were determined as the dilutions of a given immune serum that resulted in 50% reduction of RLA. The data were collected from 3 independent experiments and presented as mean±SEM in Figures or best fit values for IC_50_ with or without 95% confidence intervals calculated using Graphpad software.

### The sequences alignment and structural models

All the sequences were aligned with Maga and the structure model was made by Pymol. The numbering system of HAs used in this manuscript was based on H3 HA.

### Statistical analysis

Titration curves of PN assay were generated from data collected from the results of three independent experiments using sigmoid dose-response of nonlinear fit from GraphPad and IC_50_ along with the 95% confidence intervals were determined by the best fit values. IC_50_ values were calculated with Original software. Differences of IC_50_ values of the immune sera against different pseudotypes were analyzed with *t* test and were considered significant at *P* < 0.05.

## Results

### Comparison of the cross-clade neutralization by TH DDV, TH DDD, TH VVV, TH DV and TK DDV sera

To compare cross-H5 neutralization between heterologous and homologous prime-boost or between heterologous prime-boost with once vs. twice DNA priming, TH DDV, TH DDD, TH VVV and TH DV sera were tested against a panel of 16 HPAI H5N1 pseudotypes (Table 1) along with VSV-G pseudotype control. The H5 HA pseudotype panel covers all 10 clades, 5 subclades of clade 2 and 2 subclades of clade 7 of HPAI H5N1 viruses (S2 Table). Table 2 shows that IC_50_ value against homologous TH strain was 11,073 in TH DDV sera,2,558 in TH DV sera, 2,004 in TH DDD sera and 337 in TH VVV sera. For cross-clade neutralization, TH DDV sera neutralized all 16 pseudotypes and there were 6 high, 9 moderate and 1 low susceptible strains to TH DDV sera. TH DV sera neutralized 14 of 16 pseudotypes and there were 2 high, 10 moderate, 2 low and 2 no susceptible strains. Similarly, TH DDD sera neutralized 14 of 16 pseudotypes and there were 7 moderate, 7 low and 2 no susceptible strains. And TH VVV sera neutralized 12 of 16 pseudotypes and there were 3 moderate, 9 low and 4 no susceptible strains.

**Table 1:** The panel of HPAI H5N1 pseudotypes used in current study^a^.

Strains	Abbrev.	(Sub)clades	Strains	Abbrev.	(Sub)clades
A/Hong Kong/156/1997	HK/97	0	A/silky chicken/ Hong Kong/SF189/2001	SCk/HK/01	3
A/Thailand/(KAN-1)/2004	TH	1	A/goose/Guiyang/337/2006	Gs/GY/06	4
A/Indonesia/5/2005	ID/05	2.1.3.2	A/duck/Guangxi/1378/2004	Ck/GX/04	5
A/Turkey/65596/2006	TK/06	2.2.1	A/blackbird/Hunan/1/2004	Bb/HN/04	6
A/common magpie/ Hong Kong/5052/2007	HK5052	2.3.2.1	A/Beijing/01/2003	BJ/03	7.1
A/Shenzhen/406H/2006	SZ/06	2.3.4	A/chicken/Shanxi/2/2006	Ck/SX/06	7.2
A/chicken/Guangxi/12/2004	Ck/GX03	2.4	A/chicken/Henan/16/2004	Ck/HN/04	8
A/chicken/Korea/es/2003	Ck/KR/03	2.5	A/goose/Shantou/1621/2005	Gs/ST/05	9

^a^ All H5N1 pseudotypes expressing the same N1NA (Accession# of NA: AY555151) derived from A/Thailand/1(KAN-1)/2004.

**Table 2:** IC_50_ values of TH DDV, TH DV, TH DDD, TH VVV and TK DDV sera against a panel of H5N1 pseudotypes.

(Sub)clades	Abbrev.	TH DDV	TH DV	TH DDD	TH VVV	TK DDV
0	HK/97	15,076^a^ (13,156–17,274)	11,111 (9,083–13,596)	4,583 (4,122~5,097)	817 (713~935)	779 (667~909)
**1**	TH	**11,073**^**b**^ **(10,327–11,872)**	**2,558 (2,351~2,663)**	**2,004 (1,872~2,251)**	**337 (286~398)**	130 (112~150)
**2.1.3.2**	ID/05	1,547 (1,430–1,673)	1,127 (865–14,69)	297 (240 ~368)	104 (83~131)	2,325 (1,759~3,073)
**2.2.1**	TK/06	1,872 (1,689–2,074)	1,157 (929–1,361)	463 (435~547)	178 (145~218)	**46,382(27,701~77,700)**
**2.3.2.1**	HK5052	109 (94–127)	ND	ND	ND	ND
**2.3.4**	SZ/06	1,397 (1,260–1,548)	193 (136–247)	112 (84~150)	ND	170 (155~186)
**2.4**	Ck/GX03	845 (654–1,092)	629 (516–766)	144 (121~171)	40 (32–56)	1,611 (1,421~1,826)
**2.5**	Ck/KR/03	2,585 (2,274–2,939)	1,145 (1,052–1,203)	141 (108~182)	198 (157~251)	1,417 (1,219~1,648)
**3**	SCk/HK/01	9,766 (8,643–11,036)	3,251 (2,918–3,622)	520 (493~579)	310 (271~360)	ND
**4**	Gs/GY/06	4,608 (4,212–5,040)	2,702 (2,343–3,115)	549 (465~647)	230 (196~271)	ND
**5**	Ck/GX/04	21,057 (18,975–23,364)	9,542 (8,097–11,256)	2,311 (2,011~2,465)	1,077 (903~1,285)	191 (172~213)
**6**	Bb/HN/04	9,524 (8,606–10,543)	3,442 (2,578–4,598)	850 (636~1,134)	350 (286~424)	ND
**7.1**	BJ/03	4,034 (3,676–4,427)	321 (215~480)	125 (99–158)	ND	ND
**7.2**	Ck/SX/06	835 (801–931)	ND	ND	ND	ND
**8**	Ck/HN/04	1,795 (1,566–2,057)	1,141 (779–1,669)	359 (272~475)	81 (62–102)	ND
**9**	Gs/ST/05	12,520 (11,854–13,224)	4,554 (3,939–5,266)	2,156 (1,925~2,363)	727 (644~820)	ND
	VSVG	ND	ND	ND	ND	ND

^a^ Data collected from three independent experiments are presented and best fit values for IC50 along with the 95% Confidence Intervals in parenthesis are shown. IC50 of <500 (low) are shown in wheat, between 500 and 10,000 (intermediate) in light cyan, and >10,000 (high) in deep skyblue

^b^ Underline IC_50_ values are against the homologous strain

Interestingly, when compared with the immune sera based on TH strain, TK DDV sera induced by another H5N1 strain A/Turkey/65596/2006, whose HA sequence is further away from the consensus sequence, exhibited the highest neutralizing titer against homologous strain, but it still had the poorest cross-H5 neutralization and only neutralized 8 out of 16 strains (Table 2).

### Determination of antigenic sites (AS) recognized by TH DDD, TH VVV, TH DDV and TH DV sera

To dissect the region of HA protein recognized by the neutralizing antibodies of the immune sera[18], pseudotypes expressing head/stem chimera from TH and HK5052 HA were generated because of the low neutralizing titer of the immune sera against HK5052 (Table 2 and S2 Table). The results show that regardless of which stem was linked with, if the head region was from TH HA, high neutralization titers were detected; if the head region was from HK5052 HA, low neutralization titers were found (Table 3). This indicates that the neutralizing antibody responses of TH DDD, TH VVV or TH DV sera are also mainly directed against the HA head region.

**Table 3:** IC_50_ values of TH DDV, TH DDD, TH VVV and TH DV sera against head/stem chimera and parental viruses.

Abbrev.	TH DDV	TH DDD	TH VVV	TH DV
TH	15,755(12,407–16,536)^a^	2,194(1,883–2,556)	341(273–401)	2,401(2,101–2,743)
HK5052	151 (136–167)	40(32–51)	ND	ND
TH head HK5052 stem	15,480(12,920–16,920)	2,144(1,927–2,391)	399(362–437)	2,378(2,167–2,591)
HK5052 head TH stem	46(37–58)	48(36–55)	ND	ND

^a^ IC_50_ values collected from three independent experiments are presented and best fit values for IC_50_ values along with the 95% Confidence Intervals.

**Fig 1:**
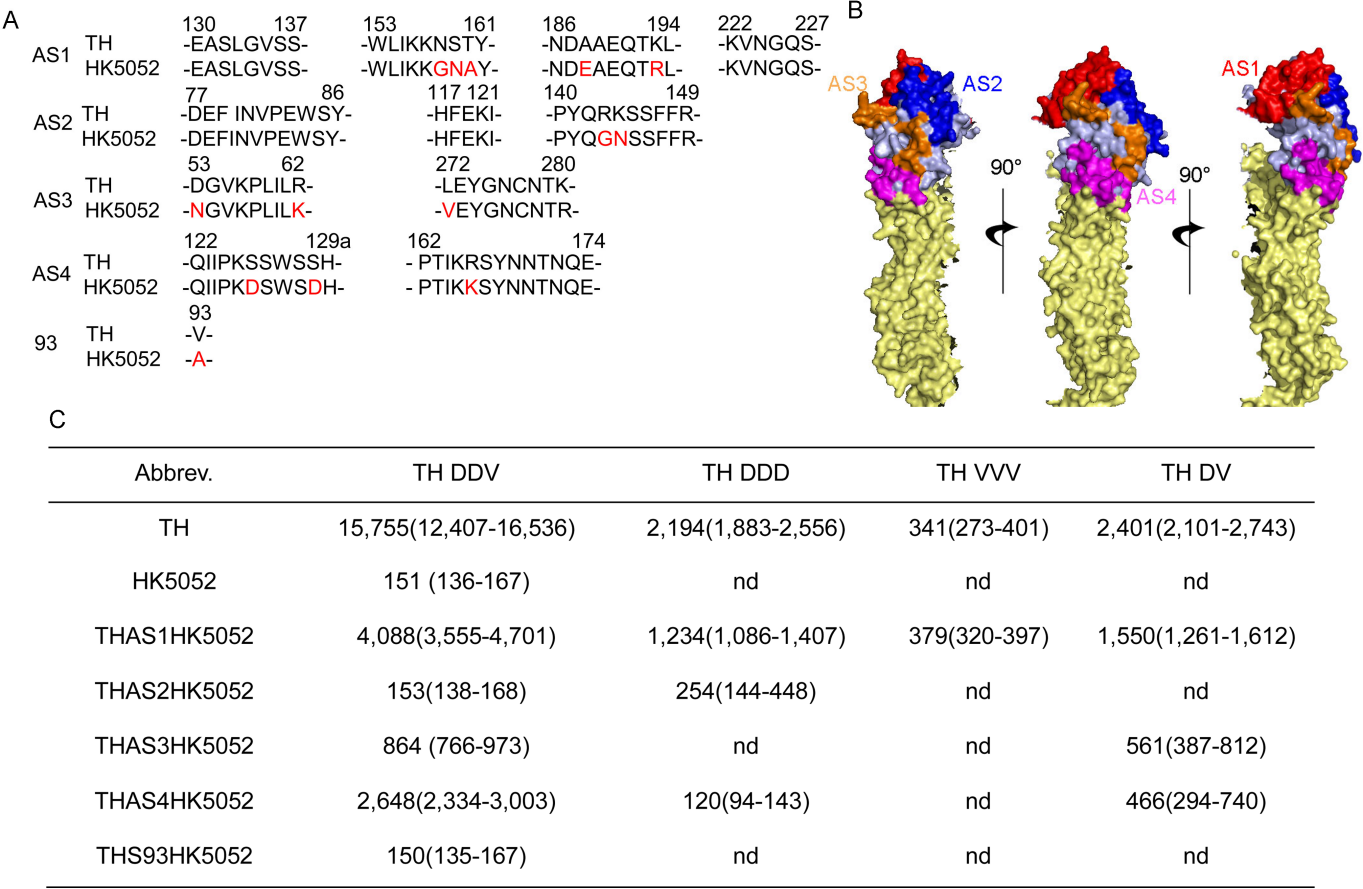
Antigenic sites recognized by TH DDV, TH DDD, TH VVV and TH DV sera. (A) Location and classification of the amino acids in the antigenic sites on TH HA and HK5052 HA. The different residues in the antigenic sites between HK5052 HA and TH HA are shown in red. (B) Structural and spatial modeling of the four antigenic sites on an H5N1 influenza strain A/Vietnam/1203/2004 HA (PDB: 2FKO). (C) IC_50_ values of TH DDV, TH DDD, TH VVV and TH DV sera against chimera and parental viruses. Data collected from three independent experiments are presented and best fit values for IC_50_ along with the 95% Confidence Intervals.

To further determine antigenic sites (AS) on TH HA head responsible for the neutralization of immune sera, we generated 5 HA chimera (THAS1HK5052, THAS2HK5052, THAS3HK5052, THAS4HK5052 and TH94HK5052), in which AS1, AS2, AS3, AS4 or amino acid residue 94 of HK5052 HA were replaced with those of TH HA (Fig 1A and 1B). The pseudotypes were generated with these HA chimera (S2 Table). Then we performed PN assays with these mutant viruses and HK5052 virus. As compared to HK5052, IC_50_ values of TH DDV sera against THAS1HK5052, THAS3HK5052 and THAS4HK5052 were significantly increased (all of *P* values were less than 0.0001), IC_50_ values of TH DV sera against THAS1HK5052, THAS3HK5052 and THAS4HK5052 were obviously increased from <40 to 1,550, 561 and 466, IC_50_ values of TH DDD sera against THAS1HK5052, THAS2HK5052 and THAS4HK5052 were obviously increased from <40 to 234, 254 and 120, and IC_50_ value of TH VVV against THAS1HK5052 was obviously increased from <40 to 379 (Fig 1C). These results indicate that both TH DDV and TH DV sera recognize AS1, AS3 and AS4, TH DDD sera recognize AS1, AS2 and AS4, and TH VVV sera only recognize AS1. Interestingly, all the immune sera recognize AS1, and the antibodies induced by AS1 contribute to the largest proportion of the neutralizing activity of the immune sera.

### Correlates between variations of the five amino acid residues in AS1 and cross-clade neutralization

To further understand the molecular basis of neutralization breadth of the immune sera, we analyzed the correlation between the amino acid residue variations in AS1 among 16 tested strains and the neutralization titers of the immune sera against these strains. The results show that the cross-clade neutralization patterns by TH DDV, TH DDD, TH VVV and TH DV sera have high correlation with variations of residues 158,159,160,188 and 193 in AS1. Based on these five residues, 16 tested strains could be divided into four groups: AK (this group contains seven strains whose residues 188 and 193 are Ala and Lys, and residues 158–160 are not Asn, Asn and Thr), AR (this group consists of five strains whose residues 188 and 193 are Ala and Arg, and residues 158–160 are not Asn, Asn and Thr), ER (this group has one strain whose residues 188 and 193 are Glu and Arg, and residues 158–160 are Gly, Asn and Ala), and NNT (this group comprises three strains whose residues 158–160 are Asn, Asn and Thr) (Table 4). The results show that the neutralization activity of TH DDV sera against the strains in AK group was high, against the strains in AR and NNT groups was moderate; and against the strain in ER group was very low. For TH DV and TH DDD sera, the neutralization of the strains in AK, AR, NNT and ER groups was high, moderate, low and undetected, respectively. The neutralization activity of TH VVV sera against the strains in AK group was moderate, against the strains in AR group was low, and against the strain in NNT and ER groups was undetected (Table 5).

**Table 4:** Residue polymorphism in AS1 at positions 158,159,160,188 and 193 among 16 strains.

Groups	Abbrev.	158	159	160	188	193	Groups	Abbrev.	158	159	160	188	193
**AK**	TH	N	S	T	A	K	**AR**	Ck/GX03	N	S	T	A	R
	SCk/HK/01	N	S	T	A	K		Ck/HN/04	N	S	T	A	R
	Gs/ST/05	N	S	A	A	K		ID/05	N	S	T	A	R
	Ck/GX/04	N	S	A	A	K		Ck/KR/03	N	S	A	A	R
	HK/97	N	S	A	A	K		TK/06	D	N	A	A	R
	Bb/HN/04	N	S	A	A	K	**NNT**	SZ/06	N	N	T	A	K
	Gs/GY/06	N	S	S	A	K		BJ/03	N	N	T	E	K
**ER**	pHK5052	G	N	A	E	R		Ck/SX/06	N	N	T	E	K

**Table 5:** Neutralization titers of the immune sera against the strains in different groups.

Groups	TH DDV		TH DV		TH DDD		TH VVV	
	Short Name	IC_50_	Short Name	IC_50_	Short Name	IC_50_	Short Name	IC_50_
**AK**	pCk/GX/04	21,057	pHK/97	11,111	pHK/97	4,583	pCk/GX/04	1,077
	pHK/97	15,076	pCk/GX/04	9,542	pCk/GX/04	2,311	pHK/97	817
	pGs/ST/05	12,520	pGs/ST/05	4,554	pGs/ST/05	2,156	pGs/ST/05	727
	pTH	11,073	pBb/HN/04	3,442	pTH	2,004	pBb/HN/04	189
	pSCk/HK/01	9,766	pSCk/HK/01	3,251	pBb/HN/04	850	pTH	337
	pBb/HN/04	9,524	pGs/GY/06	2,702	pGs/GY/06	549	pSCk/HK/01	310
	pGs/GY/06	4,608	pTH	2,558	pSCk/HK/01	477	pGs/GY/06	230
**AR**	pCk/KR/03	2,585	pCk/KR/03	1,145	pTK/06	443	pCk/KR/03	198
	pTK/06	1,872	pTK/06	1,157	pCk/HN/04	359	pTK/06	178
	pCk/HN/04	1,795	pCk/HN/04	1,141	pID/05	297	pID/05	104
	pID/05	1,547	pID/05	1,127	pCk/KR/03	141	pCk/HN/04	81
	pCk/GX03	845	pCk/GX03	629	pCk/GX03	144	pCk/GX03	40
**NNT**	pBJ/03	4,034	pBJ/03	321	pBJ/03	125	pSZ/06	ND
	pSZ/06	1,397	pSZ/06	193	pSZ/06	112	pBJ/03	ND
	pCk/SX/06	835	pCk/SX/06	ND	pCk/SX/06	ND	pCk/SX/06	ND
**ER**	pHK5052	40	pHK5052	ND	pHK5052	ND	pHK5052	ND

^a^ Data collected from three independent experiments are presented and best fit values for IC50 along with the 95% Confidence Intervals in parenthesis are shown. IC50 of <500 (low) are shown in wheat, between 500 and 10,000 (intermediate) in light cyan, and >10,000 (high) in deep skyblue.

These results suggest that the cross-clade neutralization titers of the immune sera have correlation with variations of residues 159, 188 and 193 in AS1. Substitutions of residues 158 from N to D and 160 from T to S or A have no obvious effects on the neutralization titers, but substitution of residue 159 from S to N, especially when residue 158 is N, obviously decreases the neutralization titers. Substitution of residues 188 from A to E has no obvious effects on the neutralization titers. Substitution of residue 193 from K to R obviously decreases the neutralization titers. Substitutions of residues 158,159,160,188 and 193 from NSTAK to GNAER significantly decrease the neutralization titers (Tables 4 and 5).

### Dissection of the amino acid residues in AS1

To define the effect of the residue changes in AS1 on neutralization reactivity, we generated five THAS1HK5052 mutant viruses, whose amino acid residues at positions 158,159,160,188,193 of THAS1HK5052 HA were substituted by those at corresponding positions of HK5052 HA, and tested the neutralizing activity of the immune sera against these mutants. Of the five mutant viruses, three have a single substitution at position 159, 188 or 193, one has double substitutions at positions 188 and 193, and one has triple substitutions at positions 158, 159 and 160(S2 Table).

We found that change of residue 188 from A to E does not obviously affect the neutralization titers induced by AS1, change of residue 193 from K to R obviously decreases the neutralization titers, change of residue 188 and 193 from AK to ER obvously decreases the neutralization titers, change of residues 158,159 and 160 from NST to GNA moderately affects the neutralization titers. Among three residues change of residue 159 from S to N moderately decreases the neutralization titer of TH DDV sera by 28%, but obviously decrease the neutralization titers of TH DDD sera, TH VVV sera and TH DV sera by 64%, 79% and 73%, respectively (Table 6).

**Table 6:** The neutralization of the mutants based on THAS1HK5052 by TH DDV, TH DDD, TH VVV and TH DV sera.

Short Name	TH DDV	TH DDD	TH VVV	TH DV
THAS1HK5052	4,088(3,555–4,701)^a^	1,234(1,086–1,407)	379(320–397)	1,550(1,261–1,612)
	100%^b^	100%	100%	100%
A188E	4,032(3,906–4,329)	1,148(1,024~1283)	368	1496 (1,308~1,623)
	2%	7%	3%	3%
K193R	1,936(1,635–2,117)	753(695–854)	112(95–132)	659(538–725)
	53%	49%	70%	58%
A188E, K193R	693(526–725)	339(262–432)	35(26–45)	157(123–201)
	83%	73%	90%	89%
S159N	2,960(2,241–2,679)	425(441–679)	80(64–99)	421(341–779)
	28%	64%	79%	73%
N158G,S159N, T160A	3,099(2,889–3,270)	982(839–1007)	310(282–346) 1,470)	1,279(1,097–1,470)
	24%	21%	19%	18%
HK5052	45 (35–64)	nd	nd	nd
(A188E, K193R, N158G,S159N,T160A)	99%	100%	100%	100%

^a^ IC_50_ values collected from three independent experiments are presented and best fit values for IC_50_ values along with the 95% Confidence Intervals.

^b^ Decreased percentage of neutralization against the mutants compared with parental THAS1HK5052 strain. The neutralization against THAS1HK5052 strain is regarded as 100%.

Antibodies directed to AS1 contribute to the largest proportion of the neutralizing activity of these immune sera (Fig 1C). The neutralization breadth of TH DDV sera relies on the residues variation of 188 and 193 in AS1 since the neutralization titers are dramatically affected by changes at residue 188 and 193. The neutralization breadth of TH DV, TH DDD and TH VVV mainly relies not only on the residues variations of 188 and 193 but also on the residues 159 variation, since changes of these three residues have significant effect on the neutralization titers (Tables 4–6). Thus, compared to TH DDD, TH DV and TH VVV, TH DDV approach is a very promising strategy because the neutralization breadth of TH DDV sera mainly relies on residues 188 and 193 in AS1, which are relatively conserved among 16 HA sequences.

## Discussion

In the present study we tested the neutralization breadth and potency elicited by various vaccination regimens based on an A/Thailand/1(KAN)-1/2004 H5N1 strain, whose HA sequence is the closest to the consensus sequence among diverse WHO recommended H5 vaccine strains[18]. We found that TH DDV immunization induced the broadest neutralization because this strategy elicited the most potent neutralizing titers and the neutralization breadth is positively correlated with the neutralization potency (Table 2). Moreover, compared with the various regimens based on TH strain, TK DDV sera induced by a TK strain whose HA sequence is further away from the consensus sequence exhibited the poorest cross-H5 neutralization activity, even though against homologous strain its neutralization is the highest (Table 2). This confirms that immunization with the entire HA molecule whose sequence is close to the consensus sequence can induce broad cross-H5 neutralization[18]. Thus we concluded that the combination of the immunization with the sequence close to the consensus sequence and two DNA prime plus one VLP boost could generate broad neutralizing antibodies.

A very interesting finding in the present study is that the polyclonal antibodies against the amino acid residues that locate in the classical antigenic site of HA head have broad cross-H5 neutralization activity. In general, Influenza viruses escape host immune system by changing the amino acid residues in the antigenic sites of HA protein. So the antibodies targeting the amino acid residues in antigenic sites of HA head are considered only to neutralize very limited strains. But this is not always the case. Monoclonal antibody S139/1 is a cross-reactive antibody that neutralizes multiple strains and subtypes and contacts antigenic sites A, B, and D on H3 HA head. S139/1 recognizes the key amino acid residues at positions 156,158, and 193 which are located in site B on H3 HA head [23,24]. Despite the discovery of broadly neutralizing antibodies against HA head like S139/1, C05, CH65 and 65C6, this kind of antibodies is rare isolated because HA head is very mutable[23–27]. But in the present study, similar to S139/1, large portion of broadly neutralizing antibodies elicited by TH DDV targets two key amino acid residues at positions 188 and 193 of AS1 region on H5 HA head (Table 4–6). Residues 188 and 193 locate within the 190-helix of the RBS, which corresponds to antigenic site B on H3 HA and antigenic site Sb on H1 HA [20]. Residues 188 and 193 that are relatively conserved among the 16 HA sequences contribute to the most of the neutralization activity induced by AS1 (Fig 1, Tables 4 and 6). This could explain why TH DDV sera exhibit the broad cross-H5 neutralization reactivity.

Another interesting finding is that changes of residues 188 and 193 from AK to ER of AS1 result in changes of the neutralization activity from highly susceptible to moderately susceptible (Table 6). The difference in antigenicity between AK and ER may reflect their difference in the molecular size. Side chains of ER are larger than those of AK. Changes of residue 159 from S to N obviously decrease the neutralization activity. Although residues NST at positions 158,159 and 160 are a potential glycosylation site, the structure of an H5N1 A/Vietnam/1203/2004 HA, which has only two-residues at position 44 and 143 of HA different from TH HA, shows that asparagine residue at position 158 is not glycosylated [28]. Residue N has stronger hydrophilic force than residue S because hydrophobic parameter of S are higher than that of N (N = -3.5 and S = -0.8).

We further analyze 1,663 HA sequences from different clades and subclades of H5 viruses from 2005 to 2015 available in the NCBI database. and the result shows that the amino acid residues at position 188 and 193 of 1,590 sequences can be divided into four groups: AK (36%, A at position 188 and K at position 193), EK (3%, E at position 188 and K at position 193), AR (37%, A at position 188 and R at position 193) and ER (19%, E at position 188 and R at position 193) (Table 7). We speculated that 76% (AK+EK+AR groups) of 1,663 strains are moderately to highly susceptible to the cross-H5 neutralization by TH DDV sera; 19% (ER group) of 1,663 strains are lowly susceptible; and 4.4% (neither AK, nor EK, nor AR, nor ER group) of 1,663 strains remain to be determined. This shows that although residues 188 and 193 locate in the classical antigen site, they are still relatively conserved among 1,663 HA sequences. Thus, we conclude that it is feasible to develop broad cross-H5 influenza vaccine directed to HA head.

**Table 7:** The number and percentage of different groups in 1,663 H5HA sequences.

Groups	Numbers	Percent in total number
AK	605	36%
EK	52	3%
AR	613	37%
ER	320	19%

## Supporting information

S1 Table Immunization schedule in this study. (DOCX)

S2 Table Volume, RLA and amount of HA and HIV-1 Gag p24 of all the pseudotypes added in PN assays. (DOCX)

## References

[1] Claas EC, Osterhaus AD, van Beek R, De Jong JC, Rimmelzwaan GF, Senne DA, et al Human influenza A H5N1 virus related to a highly pathogenic avian influenza virus. Lancet. 1998;351(9101):472–7. doi: 10.1016/S0140-6736(97)11212-0 9482438 10.1016/S0140-6736(97)11212-0

[2] WHO.http://www.who.int/influenza/human_animal_interface/H5N1_cumulative_table_archives/en/. 2016.

[3] WHO/OIE/FAO. Continued evolution of highly pathogenic avian influenza A (H5N1): updated nomenclature. Influenza and other respiratory viruses. 2012;6(1):1–5. doi: 10.1111/j.1750-2659.2011.00298.x 22035148 10.1111/j.1750-2659.2011.00298.x PMC5074649

[4] WHO. http://www.who.int/influenza/vaccines/virus/characteristics_virus_vaccines/en/. 2016.

[5] Pica N, Palese P. Toward a universal influenza virus vaccine: prospects and challenges. Annual review of medicine. 2013;64:189–202. doi: 10.1146/annurev-med-120611-145115 23327522 10.1146/annurev-med-120611-145115

[6] Khurana S, Loving CL, Manischewitz J, King LR, Gauger PC, Henningson J, et al Vaccine-induced anti-HA2 antibodies promote virus fusion and enhance influenza virus respiratory disease. Science translational medicine. 2013;5(200):200ra114 doi: 10.1126/scitranslmed.3006366 23986398 10.1126/scitranslmed.3006366

[7] Impagliazzo A, Milder F, Kuipers H, Wagner MV, Zhu X, Hoffman RM, et al A stable trimeric influenza hemagglutinin stem as a broadly protective immunogen. Science. 2015;349(6254):1301–6. doi: 10.1126/science.aac7263 26303961 10.1126/science.aac7263

[8] Yassine HM, Boyington JC, McTamney PM, Wei CJ, Kanekiyo M, Kong WP, et al Hemagglutinin-stem nanoparticles generate heterosubtypic influenza protection. Nature medicine. 2015;21(9):1065–70. doi: 10.1038/nm.3927 26301691 10.1038/nm.3927

[9] Kanekiyo M, Wei CJ, Yassine HM, McTamney PM, Boyington JC, Whittle JR, et al Self-assembling influenza nanoparticle vaccines elicit broadly neutralizing H1N1 antibodies. Nature. 2013;499(7456):102–6. doi: 10.1038/nature12202 23698367 10.1038/nature12202 PMC8312026

[10] Krammer F. The Quest for a Universal Flu Vaccine: Headless HA 2.0. Cell host & microbe. 2015;18(4):395–7. 26468743 10.1016/j.chom.2015.10.003

[11] Krammer F, Pica N, Hai R, Tan GS, Palese P. Hemagglutinin Stalk-Reactive Antibodies Are Boosted following Sequential Infection with Seasonal and Pandemic H1N1 Influenza Virus in Mice. Journal of virology. 2012;86(19):10302–7. PubMed Central PMCID: PMC3457330. doi: 10.1128/JVI.01336-12 22787225 10.1128/JVI.01336-12 PMC3457330

[12] Andrews Sarah F., Huang Yunping, Kaur Kaval, Lyubov., Popova, Ho Irvin Y., et al Immune history profoundly affects broadly protective B cell responses to influenza. Sci Transl Med. 2015;7(316). PubMed Central PMCID: PMCPMC4770855. 10.1126/scitranslmed.aad0522 PMC4770855 26631631

[13] Schmidt AG, Therkelsen MD, Stewart S, Kepler TB, Liao HX, Moody MA, et al Viral receptor-binding site antibodies with diverse germline origins. Cell. 2015;161(5):1026–34. PubMed Central PMCID: PMC4441819. doi: 10.1016/j.cell.2015.04.028 25959776 10.1016/j.cell.2015.04.028 PMC4441819

[14] Neu KE, Henry Dunand CJ, Wilson PC. Heads, stalks and everything else: how can antibodies eradicate influenza as a human disease? Current opinion in immunology. 2016;42:48–55. doi: 10.1016/j.coi.2016.05.012 27268395 10.1016/j.coi.2016.05.012 PMC5086271

[15] Ducatez MF, Bahl J, Griffin Y, Stigger-Rosser E, Franks J, Barman S, et al Feasibility of reconstructed ancestral H5N1 influenza viruses for cross-clade protective vaccine development. Proceedings of the National Academy of Sciences of the United States of America. 2011;108(1):349–54. PubMed Central PMCID: PMC3017181. doi: 10.1073/pnas.1012457108 21173241 10.1073/pnas.1012457108 PMC3017181

[16] Chen MW, Cheng TJ, Huang Y, Jan JT, Ma SH, Yu AL, et al A consensus-hemagglutinin-based DNA vaccine that protects mice against divergent H5N1 influenza viruses. Proceedings of the National Academy of Sciences of the United States of America. 2008;105(36):13538–43. PubMed Central PMCID: PMC2533225. doi: 10.1073/pnas.0806901105 18765801 10.1073/pnas.0806901105 PMC2533225

[17] Weaver EA, Rubrum AM, Webby RJ, Barry MA. Protection against Divergent Influenza H1N1 Virus by a Centralized Influenza Hemagglutinin. PloS one. 2011;6(3):e18314 doi: 10.1371/journal.pone.0018314 21464940 10.1371/journal.pone.0018314 PMC3065472

[18] Wang G, Zhou F, Buchy P, Zuo T, Hu H, Liu J, et al DNA Prime and Virus-like Particle Boost From a Single H5N1 Strain Elicits Broadly Neutralizing Antibody Responses Against Head Region of H5 Hemagglutinin. The Journal of infectious diseases. 2014;209(5):676–85. doi: 10.1093/infdis/jit414 23911711 10.1093/infdis/jit414

[19] Ha Y, Stevens DJ, Skehel JJ, Wiley DC. H5 avian and H9 swine influenza virus haemagglutinin structures: possible origin of influenza subtypes. The EMBO journal. 2002;21(5):865–75. PubMed Central PMCID: PMC125880. doi: 10.1093/emboj/21.5.865 11867515 10.1093/emboj/21.5.865 PMC125880

[20] Zuo T, Sun J, Wang G, Jiang L, Zuo Y, Li D, et al Comprehensive analysis of antibody recognition in convalescent humans from highly pathogenic avian influenza H5N1 infection. Nature communications. 2015;6:8855 PubMed Central PMCID: PMC4686829. doi: 10.1038/ncomms9855 26635249 10.1038/ncomms9855 PMC4686829

[21] Wei CJ, Boyington JC, McTamney PM, Kong WP, Pearce MB, Xu L, et al Induction of broadly neutralizing H1N1 influenza antibodies by vaccination. Science. 2010;329(5995):1060–4. doi: 10.1126/science.1192517 20647428 10.1126/science.1192517

[22] Zhou F, Wang G, Buchy P, Cai Z, Chen H, Chen Z, et al A triclade DNA vaccine designed on the basis of a comprehensive serologic study elicits neutralizing antibody responses against all clades and subclades of highly pathogenic avian influenza H5N1 viruses. Journal of virology. 2012;86(12):6970–8. PubMed Central PMCID: PMC3393539. doi: 10.1128/JVI.06930-11 22496212 10.1128/JVI.06930-11 PMC3393539

[23] Yoshida R, Igarashi M, Ozaki H, Kishida N, Tomabechi D, Kida H, et al Cross-protective potential of a novel monoclonal antibody directed against antigenic site B of the hemagglutinin of influenza A viruses. PLoS pathogens. 2009;5(3):e1000350 PubMed Central PMCID: PMC2652660. doi: 10.1371/journal.ppat.1000350 19300497 10.1371/journal.ppat.1000350 PMC2652660

[24] Lee PS, Yoshida R, Ekiert DC, Sakai N, Suzuki Y, Takada A, et al Heterosubtypic antibody recognition of the influenza virus hemagglutinin receptor binding site enhanced by avidity. Proceedings of the National Academy of Sciences of the United States of America. 2012;109(42):17040–5. PubMed Central PMCID: PMC3479480. doi: 10.1073/pnas.1212371109 23027945 10.1073/pnas.1212371109 PMC3479480

[25] Whittle JR, Zhang R, Khurana S, King LR, Manischewitz J, Golding H, et al Broadly neutralizing human antibody that recognizes the receptor-binding pocket of influenza virus hemagglutinin. Proceedings of the National Academy of Sciences of the United States of America. 2011;108(34):14216–21. PubMed Central PMCID: PMC3161572. doi: 10.1073/pnas.1111497108 21825125 10.1073/pnas.1111497108 PMC3161572

[26] Hu H, Voss J, Zhang G, Buchy P, Zuo T, Wang L, et al A human antibody recognizing a conserved epitope of H5 hemagglutinin broadly neutralizes highly pathogenic avian influenza H5N1 viruses. Journal of virology. 2012;86(6):2978–89. PubMed Central PMCID: PMC3302345. doi: 10.1128/JVI.06665-11 22238297 10.1128/JVI.06665-11 PMC3302345

[27] Hong M, Lee PS, Hoffman RM, Zhu X, Krause JC, Laursen NS, et al Antibody recognition of the pandemic H1N1 Influenza virus hemagglutinin receptor binding site. Journal of virology. 2013;87(22):12471–80. PubMed Central PMCID: PMC3807900. doi: 10.1128/JVI.01388-13 24027321 10.1128/JVI.01388-13 PMC3807900

[28] Stevens J, Blixt O, M T, Tumpey K J, Taubenberger, et al Hemagglutinin from an H5N1 Influenza Virus Structure and Receptor Specificity of the. Science. 2006;312:8. 10.1126/science.1124513 16543414

