# Supplementary material for: Combination of the immunization with the sequence close to the consensus sequence and two DNA prime plus one VLP boost generate H5 hemagglutinin specific broad neutralizing antibodies
Source: PLoS One. 2017 May 24;12(5):e0176854. doi: 10.1371/journal.pone.0176854 (PMC5443486; doi:10.1371/journal.pone.0176854)
Supplement: S1 Table — (DOCX) [file pone.0176854.s001.docx]

**Supplementary Table1** Immunization schedule in this study

| Groups | Day-7 | Day-0 | Day-21 | Day-35 | Day-42 | Day-56 |
| --- | --- | --- | --- | --- | --- | --- |
| TH DDV | Bleeding | TH DNA | TH DNA |  | TH VLP | Bleeding |
| TH DDD | Bleeding | TH DNA | TH DNA |  | TH DNA | Bleeding |
| TH VVV | Bleeding | TH VLP | TH VLP |  | TH VLP | Bleeding |
| TH DV | Bleeding | TH DNA | TH VLP | Bleeding |  |  |
| TK DDV | Bleeding | TK DNA | TK DNA |  | TK VLP | Bleeding |

^a^“A/Thailand/(KAN-1)/2004 strain ” is *abbreviated* to “ TH *”*, “A/Turkey/65596/2006 strain ” is *abbreviated* to “TK*”*
