# Supplementary material for: Combination of the immunization with the sequence close to the consensus sequence and two DNA prime plus one VLP boost generate H5 hemagglutinin specific broad neutralizing antibodies
Source: PLoS One. 2017 May 24;12(5):e0176854. doi: 10.1371/journal.pone.0176854 (PMC5443486; doi:10.1371/journal.pone.0176854)
Supplement: S2 Table — (DOCX) [file pone.0176854.s002.docx]

**Supplementary Table2** Volume, RLA and amount of HA and HIV-1 Gag p24 of all the pseudotypes added in PN assays

| **Strains** | **RLA** | **Volume** | **HAU of HA** | **10^5^pg of P24** |
| --- | --- | --- | --- | --- |
| A/Hong Kong/156/1997 | 249,921±21,234 | 0.010 | 4.096±0.205 | 0.037±0.002 |
| A/Thailand/(KAN-1)/2004 | 261,915±21,676 | 0.006 | 3.855±0.182 | 0.025±0.001 |
| A/Indonesia/5/2005 | 198,534±17,291 | 0.012 | 3.849±0.306 | 0.031±0.001 |
| A/Turkey/65596/2006 | 253,533±25,830 | 0.028 | 4.056±1.456 | 0.037±0.006 |
| A/common magpie/Hong Kong/5052/2007 | 225,788±13,265 | 0.037 | 4.093±1.724 | 0.023±0.004 |
| A/Shenzhen/406H/2006 | 269,509±18,154 | 0.007 | 4.055±0.191 | 0.029±0.001 |
| A/chicken/Guangxi/12/2004 | 222,324±22,563 | 0.055 | 3.986±0.606 | 0.031±0.004 |
| A/chicken/Korea/es/2003 | 218,782±29,153 | 0.110 | 3.992±1.162 | 0.024±0.002 |
| A/silky chicken/Hong Kong/SF189/2001 | 247,038±24,360 | 0.028 | 4.055±0.208 | 0.032±0.007 |
| A/goose/Guiyang/337/2006 | 211,165±18,321 | 0.110 | 3.988±0.618 | 0.031±0.013 |
| A/duck/Guangxi/1378/2004 | 248,670±18,456 | 0.028 | 4.056±0.433 | 0.034±0.004 |
| A/blackbird/Hunan/1/2004 | 246,350±21,789 | 0.023 | 3.962±0.373 | 0.027±0.002 |
| A/duck/Hubei/wg/2002 | 242,957±31,245 | 0.024 | 3.941±0.772 | 0.026±0.001 |
| A/Beijing/01/2003 | 206,006±11,563 | 0.007 | 4.055±0.319 | 0.036±0.002 |
| A/chicken/Shanxi/2/2006 | 221,137±23,145 | 0.007 | 4.055±0.269 | 0.030±0.001 |
| A/chicken/Henan/16/2004 | 244,193±25,314 | 0.010 | 4.096±0.322 | 0.035±0.001 |
| A/goose/Shantou/1621/2005 | 214,919±14,527 | 0.012 | 4.134±0.321 | 0.033±0.003 |
| HK5052 headTH stem | 228,732±23,451 | 0.012 | 3.843±0.174 | 0.040±0.002 |
| TH head HK5052 stem | 233,662±24,152 | 0.006 | 3.843±0.401 | 0.036±0.001 |
| HK5052 AS1 TH | 207,872±15,742 | 0.018 | 4.106±0.437 | 0.039±0.006 |
| HK5052 AS2 TH | 263,298±13,421 | 0.011 | 4.134±0.278 | 0.043±0.002 |
| HK5052 AS3 TH | 245,308±21,741 | 0.007 | 3.938±0.385 | 0.024±0.001 |
| HK5052 AS4 TH | 207,042±12,398 | 0.012 | 4.510±0.303 | 0.023±0.001 |
| HK5052 S94 TH | 232,137±21,021 | 0.013 | 3.992±0.291 | 0.043±0.002 |
| HK5052 AS134 TH | 196,388±12,410 | 0.016 | 4.102±0.264 | 0.038±0.001 |
| TH AS1 HK5052 | 199,162±11,240 | 0.005 | 4.161±0.082 | 0.036±0.003 |
| TH AS2 HK5052 | 213,456±20,413 | 0.007 | 3.975±0.368 | 0.039±0.001 |
| TH AS3 HK5052 | 253,956±22,851 | 0.032 | 4.027±0.800 | 0.043±0.001 |
| TH AS4 HK5052 | 288,346±25,621 | 0.006 | 4.090±0.398 | 0.042±0.001 |
| TH S94 HK5052 | 200,282±18,254 | 0.008 | 4.006±0.237 | 0.034±0.001 |
| A188E | 225,062±17,241 | 0.011 | 3.983±0.468 | 0.035±0.001 |
| K193R | 249,161±28,542 | 0.011 | 4.143±0.100 | 0.040±0.002 |
| A188E, K193R | 195,650±12,432 | 0.006 | 4.072±0.212 | 0.040± 0.001 |
| N158G,S159N, T160D | 185,499±15,624 | 0.008 | 4.141±0.420 | 0.034±0.001 |
| N158N,S159N, T160T | 222,324±18,254 | 0.055 | 3.986±0.606 | 0.031±0.004 |

^a^ Units of HA (mean±SD) in pseudotypes were determined by HA assay; amount of HIV-1 Gag p24 (mean±SD) in pseudotypes was determined by anti-HIV-1 Gag p24 ELISA kit; the volume means that to produce 200,000 RLA in PN assays, the amount of the added pseudotyes.
